# Clinical research progress on pathogenesis and treatment of Patent Foramen Ovale-associated stroke

**DOI:** 10.3389/fneur.2025.1512399

**Published:** 2025-04-11

**Authors:** Wenyao Li, Jianjun Zhang, Yier Zhang, Wuyue Shentu, Sicheng Yan, Qiulu Chen, Song Qiao, Qi Kong

**Affiliations:** ^1^Department of Special Inspection, Hangzhou TCM Hospital Affiliated to Zhejiang Chinese Medical University, Hangzhou, China; ^2^Zhejiang Hospital, Hangzhou, China; ^3^Zhejiang Chinese Medical University, Hangzhou, China; ^4^Liuzhou People’s Hospital, Liuzhou, China; ^5^Department of Neurology, Zhejiang Medical and Health Group Hangzhou Hospital, Hangzhou, China

**Keywords:** PFO-Associated Stroke, pathogenesis, acute phase treatment, secondary prevention, plugging therapy

## Abstract

Patent Foramen Ovale (PFO), a common cardiac abnormality, has been established as the most prevalent cause of Cryptogenic Stroke (CS). In 2022, the American Society of Cardiovascular Angiography and Interventions (SCAI) officially defined PFO-induced CS as PFO-Associated Stroke (PFO-AS), whose onset characteristics and treatment methods are currently the focus of pertinent clinical research. Previously, the pathogenesis of PFO-AS was commonly believed to be related to Paradoxical Embolism (PDE) or *in situ* thrombosis. Recently, atrial heart disease, which could lead to abnormal cardiac structure and circulating biomarker accumulation, potentially causing vascular endothelial injury and promoting thrombosis, has also been associated with the pathogenesis of PFO-AS. Therefore, PFO-AS could be the outcome of multiple pathogenesis mechanisms. Furthermore, significant research progress has been made in elucidating the pathogenic PFO gene. Nonetheless, additional in-depth research is still required to better elucidate the precise mechanisms underlying PFO-AS. Notably, the clinical and imaging characteristics of PFO-related Ischemic Stroke (IS) are slightly different from those of other IS causes. Furthermore, the assessment of the correlation between PFO and stroke mostly relies on The Risk of Paradoxical Embolism Score (RoPE) and PFO-Associated Stroke Causal Likelihood classification (PASCAL) system, which could be a limitation. Additionally, PFO examinations mainly relied on cardiac anatomy evaluation in the past, highlighting another potential gap. Moreover, recent research suggests that PFO closure may increase the risk of Heart Failure (HF) with preserved Ejection Fraction (HFpEF). Conversely, after 2017, four Randomized Controlled Trials (RCTs): CLOSE, RESPECT, REDUCE, and DEFENSE-PFO, demonstrated that transcatheter PFO closure is more effective in preventing various risk events than conventional pharmacotherapy. This review comprehensively summarizes the latest research progress on PFO-AS pathogenesis, treatment, prevention, and management decisions, providing a valuable clinical reference.

## 1 Introduction

Cryptogenic Stroke (CS) is a type stroke that cannot be linked to a definitive cause after evaluating all known possible causes or has an unknown cause despite examinations. Notably, CS accounts for 25% of all Ischemic Stroke (IS) cases, and its severe nature increases the risk of death, disability, and recurrence, placing a huge burden on medical care systems and society as a whole. The most common causes of CS include Patent Foramen Ovale (PFO), Paroxysmal Atrial Fibrillation (PAF), latent arrhythmia, latent malignant tumor, and so on. As delineated within the confines of a literature about “NeuroVISION study” (1), individuals harboring a PFO, when contrasted with those perioperative stroke patients devoid of such an anomaly, exhibit an elevated risk of stroke, augmented National Institutes of Health Stroke Scale (NIHSS) scores, and a higher incidence of in-hospital mortality. This corroborates the assertion that the PFO constitutes a significant risk factor for stroke. In CS patients, the PFO detection rate is 40–56%, significantly higher than that in healthy individuals (4–18%) and other stroke patients with clear causes, potentially leading to serious complications (2). Given its significant prevalence, in 2022, the American Society for Cardiovascular Angiography and Interventions (SCAI) officially defined PFO-induced CS as PFO-Associated Stroke (PFO-AS) (3). Significant advancements in PFO-AS research have also been recently realized, especially from 2017, when the New England Journal of Medicine (NEJM) published the research results of CLOSE (4), REDUCE (5), and RESPECT (6). These studies reported that PFO closure surgery can reduce the risk of stroke recurrence more effectively than conventional drug therapy, greatly informing PFO-AS treatment and secondary prevention. Nonetheless, more insights into the prevention and treatment of PFO-AS are still required, making PFO-related research highly relevant contemporary research topic. There is also a need to further explore the pathophysiological mechanisms underlying PFO-AS. This review will focus on PFO-AS pathogenesis, diagnosis, treatment, and prevention, providing a valuable reference for other related studies and clinical management.

## 2 Pathogenesis

**Figure 1:**
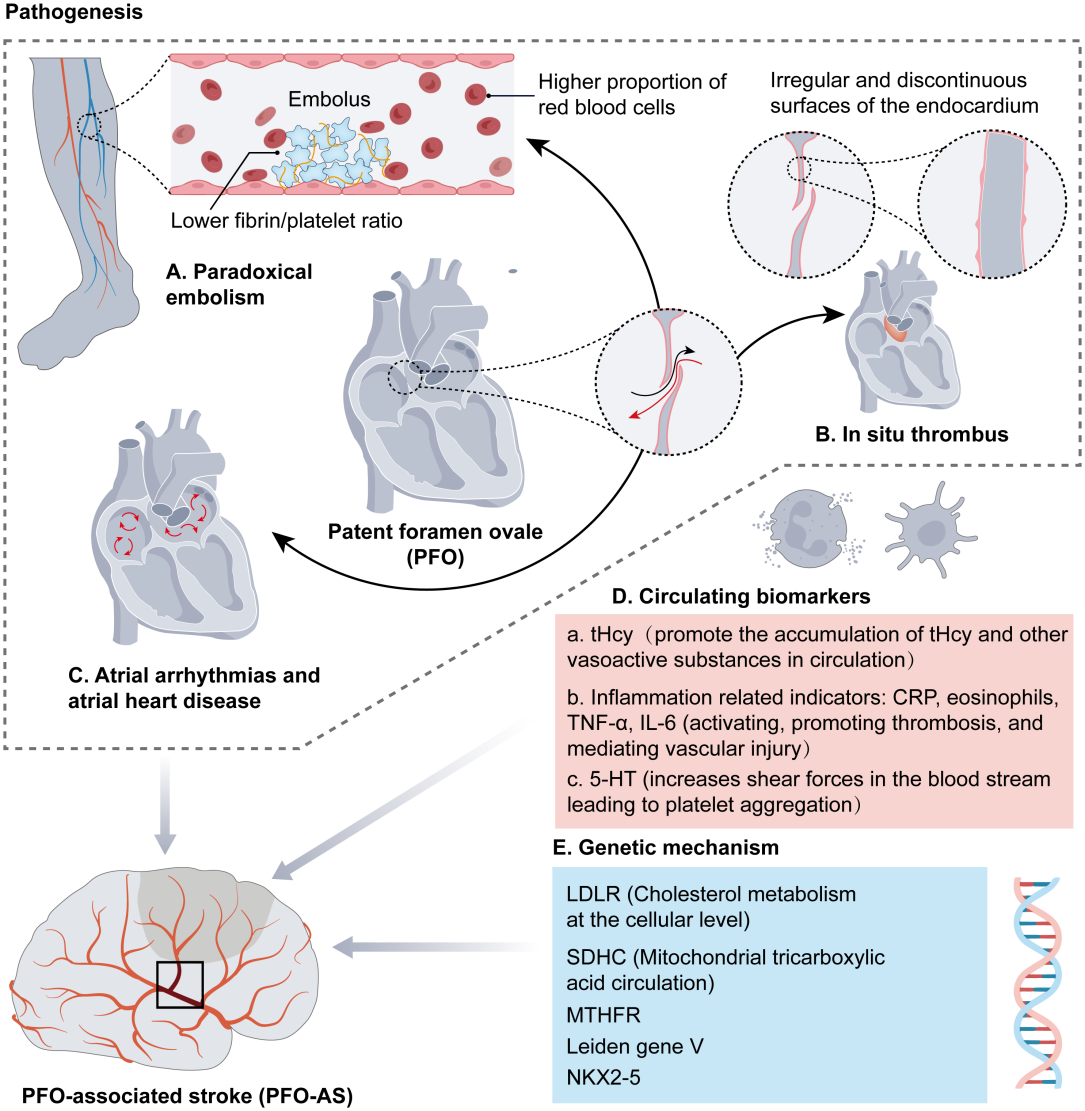
Pathogenesis of PFO-AS.

The foramen ovale is a physiological channel in the heart’s atrial septum that develops in the embryonic stage. Normally, this channel closes between 5 and 7 months post-birth. Notably, this channel would be referred to as PFO if it remains open after 3 years of age. Recently, increasing research has closely linked PFO with CS, implying the potential involvement of Paradoxical Embolism (PDE), which increases the risk of other related diseases, thus threatening patients’ lives (Figure 1).

### 2.1 Paradoxical embolism

The PDE concept was first proposed by Julius Cohenheim, a German pathologist, in 1877 (7). It occurs when patients with Venous Thromboembolism (VTE) have a right-to-left Shunt (RLS) channel. In such situations, emboli in the systemic venous system or right heart pass to the left or systemic arterial system via other arteriovenous pathways, such as the open foramen ovale, causing embolism, which, in turn, leads to ischemic infarction at the corresponding site or organ. This phenomenon is currently considered the primary etiological mechanism of PFO-AS (8).

Ohanna Härtl et al. (9) performed mechanical thrombectomy on 58 CS patients and then histologically examined the retrieved thrombi to further elucidate the pathophysiological mechanisms underlying PFO-AS. According to the results, PFO-AS patients had a higher proportion of Red Blood Cells (RBCs) and a lower fibrin/platelet ratio in the thrombus component compared to patients with CS alone, implying that PFO-related thrombi may originate in the venous system, resulting in IS via PDE. However, they could only detect deep vein thrombus in 1.1 to 27% of PFO-AS patients and could still not identify the source of the emboli in 80 to 90% of PFO-AS patients (10). These findings suggest the involvement of other mechanisms in PFO-associated thrombus besides PDE.

### 2.2 *In situ* thrombus

Generally, the left atrial pressure is often higher than the right atrial pressure, hence, the PFO is often closed and accompanied with no significant RLS production. When patients perform the Valsalva maneuver (breath-holding and forced expiration maneuver following deep inspiration), cough, or sneeze, because the pressure gradient may not be large enough, the blood flow in the PFO tunnel is low-velocity or even stasis, allowing platelets to easily adhere, aggregate, and activate, resulting in thrombus formation *in situ*. In this regard, some scholars have questioned whether the foramen ovale is the source or channel for thrombus formation. For instance, in a preliminary study on PFO *in situ* thrombus formation involving stroke patients with PFO and non-stroke patients, researchers at Fuwai Hospital first assessed the microstructure of the foramen ovale via high-resolution Optical Coherence Tomography (OCT) (11). According to the results, 11 stroke patients and one non-stroke patient with a migraine exhibited thrombus *in situ* within the PFO. Furthermore, all 12 patients presented with irregular or discontinuous endocardial surfaces, implying that endocardial abnormalities at the PFO could be the pathological basis for PFO-associated *in situ* thrombus. Although this study’s sample size was small, the *in situ* thrombus detection rate was as high as 100%, and all patients exhibited abnormal endocardial alterations. These findings are consistent with those of Kasner SE et al., which suggested that endocardial abnormalities could be responsible for thrombus *in situ* (12). Furthermore, in Yan et al.’s cross-sectional study that was published in Strokein 2023 (13), 131 PFO patients with unknown risk factors were divided into three groups: stroke, migraine, and asymptomatic. Subsequently, OCT was used to assess the incidence and size of primary thrombus in the groups of PFO patients. According to the results, compared to the asymptomatic group (which showed no *in situ* thrombus), the stroke and migraine groups showed a significantly higher incidence of *in situ* thrombus. This study further confirms that *in situ* thrombus could be a characteristic feature of PFO-AS patients and migraine patients, there are important implications for guiding treatment. Overall, endocardium abnormalities at the foramen ovale are common in patients with *in situ* thrombus; thus, thrombus attached at these irregular and discontinuous surfaces of the endocardium is highly likely to be associated with *in situ* thrombus formation.

Although the above-mentioned studies on *in situ* thrombus had small sample sizes and OCT technology could be vulnerable to visual field, it is noteworthy that *in situ* thrombus, as a mechanism, may represent a novel potential therapeutic target for antithrombotic treatment or prophylactic PFO closure in PFO-AS patients.

### 2.3 Atrial arrhythmias and atrial heart disease

Atrial heart disease denotes a condition where alterations in the structure, systolic function, or electrophysiological properties of the atrium result in clinical manifestations like atrial remodeling and conduction abnormalities. It is often suspected to be an underlying cause of unexplained embolic stroke, independent of atrial fibrillation (14). Atrial arrhythmias refer to abnormal electrical activity originating from the atrium, leading to irregular heart beat frequency or rhythm.

PFO, a physiological opening in the atrial septum, typically bears no direct causal relationship with atrial arrhythmias. However, in cases of certain structural heart diseases, such as atrial septal aneurysms (ASA), both PFO and atrial arrhythmias may coexist. Studies indicate (15) that when blood flow within the atrium enters the ASA, the velocity slows down, creating eddies. This can not only lead to blood stasis and subsequent thrombosis but also continuously stimulate the atrial electrophysiological conduction system, ultimately triggering atrial arrhythmias. In addition, if PFO patients exhibit a significant number of RLS, on the one hand, the abnormal blood flow may stimulate the atrial electrophysiological system, subsequently triggering ectopic atrial electrical activity, increasing the likelihood of atrial arrhythmias. On the other hand, hemodynamic changes can elevate atrial pressure or volume load and ultimately progressing to atrial heart disease. Under such circumstances, blood flow tends to stagnate, increasing the risk of thrombosis, which in turn heightens the occurrence of adverse cardiovascular events, including stroke (16, 17). Recent studies have further corroborated the mechanism behind blood stasis and thrombosis in PFO patients. In 2023, researchers conducted a specific 3D computational fluid dynamics analysis to compare the differences in blood retention time within the left atrium among patients with PFO, atrial fibrillation, and those with normal heart rhythm. This comparison aimed to assess the extent of left atrial blood stasis. The findings revealed that patients with PFO and those suffering from atrial fibrillation exhibit analogous blood flow patterns, as well as structural and functional abnormalities in the left atrium. These abnormalities heighten the incidence of atrial electrophysiological abnormalities, thereby increasing the risk of stroke (18).

### 2.4 Circulating biomarkers

Research has shown that small molecule metabolites are also involved in the pathogenesis of cardiovascular and cerebrovascular illnesses (19).

#### 2.4.1 Homocysteine (Hcy)

Whether by administering exogenous homocysteine *in vitro* or through ex vivo blood tests and animal models involving patients with elevated homocysteine levels, current research findings suggest that Hcy can induce oxidative stress, triggering platelet activation, hypercoagulable state formation, endothelial dysfunction, and influencing the degree and speed of blood clot contraction during the thrombosis process, ultimately promoting thrombus formation (20).

Deng et al. (17) prospectively examined PFO-AS patients and continuously performed the serial sampling of cardiac, atrial, and venous blood before and after PFO closure using Mass Spectrometry (MS) analysis. Specifically, they aimed to explore the effect of occlusion on circulation in PFO patients. According to the results, Hcy was the most significantly downregulated factor in intracardiac plasma after PFO closure, correlating positively with the number of PFO shunts. Furthermore, during the four-year follow-up period, Hcy levels in venous blood were lower following complete PFO closure compared to cases intervened with medical treatment alone (including antiplatelet agents and anticoagulants). Notably, Hcy levels did not change in patients who received medical treatment alone. These findings offer a major reference for molecular clinical research, demonstrating that PFO-induced shunting, especially large-sized shunting, could promote the accumulation of Hcy and other vasoactive substances in circulation. Overall, besides being a channel through which blood clots pass, PFO could also lay the structural foundation for thrombus formation, causing damage to the nervous and blood systems.

#### 2.4.2 Inflammation-related indicators

According to research (21), C-Reactive Protein (CRP) upregulation is an independent risk factor for IS events. In addition, proinflammatory cytokines such as Tumor Necrosis Factor-*α* (TNF-α) and Interleukin-6 (IL-6), besides being involved in the inflammatory response of IS, can activate endothelial cells’ normal anticoagulant and fibrinolytic properties, promoting thrombus formation, which could, in turn, lead to venous thrombosis and stroke (22). Moreover, retrospective studies reported that eosinophils could activate and promote thrombus formation, as well as mediate vascular injuries. Specifically, patients with RLS exhibited a higher proportion of eosinophils in whole blood than those without RLS. Additionally, the proportion of eosinophils correlated positively with shunt volume, indicating an association between a greater shunt volume and an increased presence of eosinophils (23). Based on these findings, we deduced that eosinophils may be involved in the development of stroke via vascular injury mediation and thrombus activation and induction, leading to venous microthrombi entering the left atrium through the foramen ovale.

#### 2.4.3 Serotonin (5-Hydroxy tryptamine, 5-HT)

5-HT is a vasoactive prothrombotic substance that induces Oxidative Stress (OS) within the heart. Generally, 5-HT is released from aggregated platelets, remains in venous blood, and is metabolically inactivated by pulmonary Monoamine Oxidase (MAO) in RLS absence. Notably, PFO presence increases shear forces in the bloodstream leading to platelet aggregation, thus facilitating the release of significant amounts of 5-HT. At the same time, 5-HT free in blood could further promote platelet aggregation, damage vascular endothelial cells, promote thrombus, and increase the risk of stroke (21).

Overall, PFO presence directly or indirectly lays the pathophysiological foundation for thrombus formation, and the accumulation of circulating small molecule metabolites could alter the status of normal endothelial cells, not only increasing the risk of PDE in PFO patients but also directly promoting thrombus formation, mediating vascular injury, and increasing the risk of stroke.

### 2.5 Genetic mechanism

Genetic factors have been established to significantly influence CS as a severe PFO outcome. According to research (24), coagulation-associated genetic variants, genetic susceptibility to cardio-structural abnormalities, and genetic variants associated with arterial wall elasticity and stability highlight the significance of genetic factors in PFO-AS. Xinyi Li et al. (25), in their 2024 study, employed Whole-Exome Sequencing (WES), a gene sequencing technology, to identify the potentially mutated genes and gene mutation spots in PFO patients before analyzing the PFO-associated mutated genes using ClinVar and OMIM databases. According to the results, stroke occurred in 3 of the 25 PFO patients enrolled in the study, with a suspected causative genetic variant (LDLRNM 00527.5c947A > G) detected in one patient. Notably, this causative genetic variant has been associated with familial hypercholesterolemia and could increase the risk of cardiovascular and cerebrovascular diseases. After subjecting all 25 patients with clinical symptoms and undergoing PFO closure to WES, Xinyi Li et al. also discovered mutations in 48 related genes, of which LDLR and SDHC were suspected to be pathogenic genes. The LDLR and SDHC genes are primarily involved in cholesterol metabolism at the cellular level and mitochondrial Tricarboxylic Acid circulation (TCA), respectively. Through the detection of enrolled cases and database analysis, the researcher s also discovered that the NKX 2–5 gene was involved in PFO pathogenesis through other mutation sites and signaling pathways. This finding is consistent with the 2016 study by Cao Y et al., which suggested that variations at the NKX 2–5 single nucleotide site could be associated with atrial septal defects (26). Moreover, two recently published case reports (27) found that mutations in the MTHFR gene or heterozygous mutations in the V Leiden gene correlated with morbidity in young PFO-AS patients. According to previous research (28), 33.3% of heterozygous mutation carriers of the Leiden gene V developed varying degrees of thrombus, suggesting that heterozygous mutation of the Leiden factor V correlates significantly with the prothrombotic state. This finding is consistent with the meta-analysis results of Alhazzani et al. (5, 29). Overall, identifying PFO-associated genetic variants via genetic testing enables the early detection of high-risk PFO patients, as well as timely reduction and intervention of complications, especially illnesses with a high risk of adverse outcomes such as CS. Moreover, the study of causative and mutated genes in PFO-AS patients could yield novel ideas for genetic counseling and gene therapy in PFO-AS patients.

As opposed to past clinical research that mainly focused on PDE regarding the pathogenesis of PFO-AS, *in situ* thrombus, atrial arrhythmias, atrial heart disease, circulating biomarkers, and causative genes, have recently gained significant research attention in the same context. In this regard, it is noteworthy that more scholars have attributed the pathogenesis of PFO-AS not to a single factor but to a myriad of clinical variables. Nonetheless, additional studies with larger samples will be required in the future to further explore the relevant biochemical parameters or even genomics to better elucidate the mechanisms underlying PFO-AS and facilitate its early diagnosis, thus guiding treatment and prognostic evaluation.

## 3 Criteria for diagnosis

### 3.1 General conditions

Although PFO-AS is generally comparable to CS, its incidence rate was found to be higher in males than females, potentially due to the influence of estrogen in women, which could increase the protective effects of cerebral blood flow on the nervous system via mechanisms such as antioxidant free radicals. Furthermore, a previous study that involved women aged ≥53 years found no significant gender difference among PFO-AS patients, potentially due to the fact that 90% of Chinese women reach menopause at the age of 53 (30). From an age distribution perspective, PFO-AS was more prevalent among young and middle-aged individuals (mean age = 46.59 ± 11.732 years), with a statistically significant difference compared to patients with CS without PFO. Previous research (31) has also identified younger age at onset as a significant risk factor in assessing PFO-AS, a phenomenon that was verified using The Risk of Paradoxical Embolism Score (RoPE) results. In other words, the younger the age, the higher the score, and the more likely it was to diagnose PFO-AS. Regarding neurological impairment, PFO-AS patients had median NIHSS and Modified Rankin Scale (mRS) scores of 2 (1,4) and 1 (1,2), respectively. These values were comparable to those of CS patients without PFO. Furthermore, there was no statistically significant difference between the two groups in terms of PFO size. The average PFO diameter among the patients was 4.9 mm (1–19 mm), which could allow thrombi to pass through, although it was less likely to completely block the main blood flow. Additionally, although brain tissue damage accumulated in PFO-AS patients, the extent of neurological impairment was relatively mild. Regarding pre-admission Blood Pressure (BP), for CS patients, it gradually rose from stroke onset, peaking upon admission. Regarding previous diseases, PFO-AS patients most likely had a smoking history and Hypertension (HTN).

### 3.2 Scores for assessing PFO-AS

The RoPE score and PFO-Associated Stroke Causal Likelihood classification (PASCAL) system are the most commonly used approaches for assessing the association between PFO and stroke. The higher the score, the greater the likelihood of PFO-AS (32), especially when considered alongside clinical manifestations and imaging results. PASCAL system uses the PASCAL score to assess the likelihood of PFO-related stroke (2). The likelihood of PFO causing stroke is evaluated based on the presence of high-risk PFO morphological features (large RLS or ASA) on TEE and whether the RoPE score is ≥7 points. The results can be classified as unlikely, possible, and probable. Currently, a RoPE score > 6 is defined as PFO-AS (or stroke of other causes). Notably, the RoPE score is a vital component of the PASCAL system, which helps with further classification (Tables 1, 2) (32).

**Table 1:** RoPE score.

Characteristic	Points
RoPE score calculation	
No history of	
Hypertension	1
Diabetes	1
Stroke or transient ischemic attack	1
Nonsmoker	1
Cortical infarct on imaging	1
Age, y	5
18–29	4
30–39	3
40–49	2
50–59	1
60–69	0
>79	
Total Rope Score (sum of individual points)=	

**Table 2:** PASCAL system.

High rope score (≥7)	High-risk PFO feature (LS and/or ASA)	PFO-related stroke
Absent	Absent	Unlikely
Absent	Present	Possible
Present	Absent	
Present	Present	Probable

### 3.3 Imaging features

For PFO-AS patients, cranial Magnetic Resonance Imaging (MRI) could yield relevant and specific outcomes. First, imaging often reveals multiple infarcts in various vascularized areas, with a predominance of posterior circulation infarcts. Furthermore, posterior cerebral artery territory infarcts are independent predictors of PFO presence in CS patients. These phenomena are generally consistent with those of Kim et al. (33) who earlier proposed that PFO strokes often manifest as multiple small ischemic lesions in the vertebrobasilar circulation without any visible vessel occlusion on angiography. Second, on T2W or DWI imaging, more infarcts often present as multiple cortical lesions, with fewer lesions in the subcortical and cortico-subcortical regions. Furthermore, it was previously established that small and medium-sized lesions were more frequent and highly likely to affect posterior circulation during acute or recurrent stroke (34). These phenomena could be attributed to the fact that epinephrine weakly regulates the vertebrobasilar artery, and blood flow increases when performing the Valsalva maneuver, thus increasing the probability of thrombus entering the posterior circulation. Therefore, when the above-mentioned characteristic imaging findings appear in cranial MRIs, a PFO should be highly considered, and the underlying cause could be established based on the imaging findings.

### 3.4 PFO-associated tests

As per the relevant guidelines (35), PFO-related tests encompass a variety of procedures, including Transthoracic Echocardiography (TTE), Enhanced Transcranial Doppler Ultrasound (c-TCD), Transcatheter Echocardiography (TEE), Transductal Sonography (c-TEE), cardiac ultrasonography, and intracardiac echocardiography. Notably, c-TEE and intracavitary ultrasound are often used to diagnose and treat special cases.

Presently, TTE and TEE are the commonly used approaches for diagnosing PFO, with both methods having the capability of evaluating the anatomy of the interatrial septum and the interatrial septal shunt. More specifically, TTE could be used to directly observe the atrial septal discontinuity signal and blood flow signal by placing the probe on patients’ chest walls. Previous research (36) showed a sensitivity of 50% for TTE. On the other hand, Mojadidi et al. (37) found a 93% specificity in CS patients using harmonic imaging. Although TTE offers high specificity, it is noteworthy that obesity and emphysema, among other risk factors, could affect its performance in adults, potentially resulting in a relatively low sensitivity. Moreover, since TEE can more directly observe the internal structures of the heart through the esophagus and display the fusion of primary and secondary septa, as well as detect fine shunts and classify PFO, it could offer higher sensitivity and specificity compared to TTE (38, 39).

The c-TCD test is a standard method for detecting the presence or absence of RLS. Using the c-TCD test, RLS could be quantified by observing the number of bubbles at rest and after the Valsalva maneuver. For RLS, the c-TCD test has sensitivity and specificity values of 65–100% and 97–100%, respectively. According to related research (40), c-TCD could also confirm the presence of smaller PFOs by monitoring blood flow patterns during a stronger Valsalva maneuver. Consequently, c-TCD can detect 90–100% of PFOs identified by TEE and even find small PFOs that TEE misses in some cases. Based on these insights, c-TCD is a useful tool for clinically screening PFO and is the most convenient and highly accepted noninvasive test for patients. However, it is difficult to determine the source of RLS using c-TCD.

Overall, improving the detection rate of PFO-AS based on patients’ general conditions, clinical manifestations, RoPE score, PASCAL system, and imaging findings could yield a more accurate treatment plan for future interventions.

## 4 Treatment

In treating definite IS, it is noteworthy that PFO-AS has the same therapeutic principles as acute IS. Acute IS treatment primarily involves monitoring patients’ vital signs, opening the infarcted vessel as soon as possible, restoring intracranial perfusion, protecting brain tissue nerve function, and promptly addressing complications. While monitoring patients’ basic vital signs, recanalization therapy is the key intervention during the acute phase, and it mainly includes intravenous thrombolytic therapy, Endovascular Treatment (EVT), and antithrombotic therapy.

### 4.1 Acute phase treatment

#### 4.1.1 Thrombolytic therapy

In consonance with the directives of the American Heart Association/American Stroke Association (AHA/ASA) (41), the enhancement of cerebral circulation constitutes a critical therapeutic modality. It is imperative for patients who have undergone brain imaging diagnostics and present with an onset time of 4.5 h or less to receive intravenous thrombolytic therapy expeditiously. It is underscored that the administration of intravenous alteplase is associated with significant therapeutic benefits. According to research, PFO-AS patients treated with alteplase within the intravenous thrombolysis window may exhibit a better prognosis than stroke patients of other etiologies (42). On the one hand, rt-PA could exert a notable dissolution effect if the primary underlying cause of PFO-AS is PDE, where the emboli originate from the venous system. In such cases, the detached emboli often consist primarily of fibrin and RBCs and are loose in texture, thus resulting in a high recanalization rate. On the other hand, patients who receive rt-PA intravenous thrombolysis and are younger at the onset of stroke tend to have a better prognosis. These deductions are consistent with the previous conclusion proposed by Gaffney PJ (43), Schwartz ML (44), and other scholars that in acute stroke patients with RLS, since the emboli originate from a fibrin-rich thrombus in the deep venous system, symptoms may improve more after rt-PA thrombolysis. It is also noteworthy that the use of third-generation thrombolytic drugs such as Tenecteplase in PFO-AS was recently explored further (45). Furthermore, in 2024, Ruixian Wang published an article in Neurologist (46), reporting that applying recombinant human urinary kininogen following intravenous thrombolysis greatly improved neurological function and reduced stroke recurrence in acute IS patients. In another study, Haiqing Song et al. (47) discovered no increase in the mortality rate or risk of symptomatic intracerebral hemorrhage in >60% of patients over a time window of 4.5–6 h, implying that intravenous recombinant human prourokinase was effective and safe in patients within 4.5–6 h after stroke onset. Given that multiple studies have confirmed that the use of recombinant human prourokinase after intravenous thrombolysis could effectively improve the safety of thrombolysis and late neurological recovery, then the therapeutic results for PFO-AS patients are equally promising.

#### 4.1.2 Mechanical thrombectomy

Some of the common EVT interventions include mechanical thrombectomy, intra-arterial thrombolysis, and angioplasty. In recent years, EVT, especially mechanical thrombectomy, has become a vital treatment for IS patients, particularly those with a large vessel occlusive stroke, who are beyond the thrombolysis time window and with contraindications to thrombolysis. According to relevant research (48), compared to pharmacotherapy, mechanical thrombectomy resulted in a higher recanalization rate and a better neurological prognosis in stroke patients with large vessel occlusion of anterior circulation within 6 h post-onset. Consequently, many centers have extended EVT to large vessel occlusions in the posterior circulation, particularly basilar artery embolization (49). Previous research has also revealed that imaging is characterized by multiple infarcts in poly vascularized areas and posterior circulation infarcts, as well as posterior cerebral artery territory infarcts, which are independent predictors of PFO presence in CS patients. Moreover, recent RCTs (50, 51) and meta-analyses (52–55) on acute IS patients demonstrated that concomitant medical therapy with EVT is more effective than medical therapy alone in obtaining good functional outcomes. At the same time, some studies with small sample sizes posited that combined EVT for basilar artery occlusion could yield better outcomes (56–58). Consequently, we hypothesized that PFO-AS patients, especially those with lesions accumulated in the posterior circulation, may benefit more from EVT, a deduction that certainly requires further validation through prospective RCTs (59). Nonetheless, it is noteworthy that vascular injury (perforation, dissection, or pseudo aneurysm) could complicate EVT, and a more professional and precise assessment would be required for the risk of vasospasm (60).

#### 4.1.3 Drug therapy

Current medical interventions for acute IS mainly include antiplatelet and anticoagulant therapies. Furthermore, aspirin or clopidogrel treatment should be initiated as early as possible for patients who do not satisfy the criteria for intravenous thrombolysis or do not require EVT. For patients with mild stroke, dual antiplatelet therapy (DAPT), consisting of aspirin and clopidogrel, is recommended within 24 h of stroke onset and should be continued for 21 days (61). On the other hand, for mild stroke patients carrying CYP2C19 loss-of-function alleles, aspirin combined with ticagrelor is recommended and should be continued for 21 days (62).

A recent ARAMIS trial published in JAMA found (63) that aspirin plus clopidogrel was no less effective compared with intravenous thrombolysis in the management of non-disabling minor stroke. Clinically, PFO-AS is prevalence in young adults (64) and is associated with relatively low disability (31, 65–67). The classification of PFO-AS as a non-disabling minor stroke warrants further investigation. Moreover, DAPT may serve as an alternative to thrombolytic therapy if appropriate assessment criteria are established for PFO-AS. Compared to thrombolytic therapy, patients treated with dual antibodies exhibited lower rates of bleeding and other complications. Zi W et al. (68) subsequently conducted a randomized controlled study at 117 centers in China to investigate the effect of tirofiban, a class of glycoprotein (GP) IIb/IIIa receptor antagonist, in patients with moderate to severe disability and no macrovascular occlusive stroke. The study showed that 29.1 and 22.2% of patients in the tirofiban and aspirin groups, respectively, achieved good functional prognosis; Moreover, the incidence of symptomatic cerebral hemorrhage in the tirofiban group was slightly higher than that in the aspirin group (1.0, 0%), and there was no significant difference in mortality between the two groups (3.8, 2.6%). In recent years, clinical studies have demonstrated that only about 10% of patients can achieve early recanalization of occluded vessels following intravenous thrombolysis. Therefore, adjuvant drug therapy should be administered to improve the efficacy of intravenous thrombolysis (69). Another RESCUE-BT2 study by Zi W et al. (68) showed that the proportion of patients treated with tirofiban who achieved good prognosis was significantly higher compared with those who received aspirin in the subgroup of patients without neurological improvement after intravenous thrombolysis. However, given the small sample size of this study, larger randomized controlled studies are needed to validate the safety and efficacy of intravenous thrombolysis combined with tirofiban treatment. Elsewhere, a trial named, (Multi-arm Optimization of Stroke Thrombolysis; ClinicalTrials.gov number, NCT03735979) randomized controlled study investigated the efficacy and safety of eptifibatide, a glycoprotein IIb/IIIa receptor antagonist, and argatroban, an anticoagulant, combined with alteplase thrombolysis in patients with ischemic stroke.

In patients with acute ischemic stroke, emergency thrombolytic therapy administered within 0–3 h can improve the symptoms of neurological deficits, promote motor function recovery and self-care ability, and reduce adverse reactions compared with administration between 3 and 5 h. In clinical practice, few patients can achieve thrombolysis within 3 h, especially the aged groups. Thrombolysis is not only affected by objective factors such as detection time, transport time, and examination time, as well as the cognitive understanding of the patient and his family about the condition.

### 4.2 Secondary prevention

#### 4.2.1 Drug therapy

Secondary prevention of PFO-AS is increasingly being studied worldwide. All PFO-AS patients, regardless of whether they receive thrombolysis or intervention, should be administered antithrombotic therapy. The PICSS (CS with PFO) study found no significant reduction in stroke recurrence or death 2 years after treatment with warfarin compared with aspirin (70). In the PFO subgroup of the rivaroxaban Secondary Prevention for Unexplained Embolic Stroke Study (NAVIGATE ESUS) (71), the annual stroke risk was 2.6 and 4.8% in the rivaroxaban and aspirin groups, respectively, and rivaroxaban was slightly superior to aspirin in decreasing the risk of recurrent ischemic stroke. Diener et al. (72) performed a meta-analysis of patients who developed PFO-AS in four trials, PICSS (70), CLOSE (4), NAVIGATE ESUS (71), and RESPECT (6), and reported that the risk of recurrent ischemic stroke was similar between anticoagulant and antiplatelet therapy groups. Given the predominantly venous origin of thrombi in PFO-AS and the associated risk of bleeding with anticoagulation, antiplatelet therapy is often considered a more appropriate secondary preventive measure. However, anticoagulation is recommended for PFO-AS patients with pulmonary embolism, deep vein thrombosis, or underlying hypercoagulable states (73).

#### 4.2.2 Plugging therapy

##### 4.2.2.1 Applicable conditions for PFO closure

In accordance with the 2022 SCAI Guidelines for Management of Patent Foramen Ovale (3), for individuals aged between 18 and 60 years, who lack anticoagulation indications, possess high-risk anatomic configurations, and have a RoPE score of 7 or higher, and have a history of PFO-related stroke, PFO occlusion may be considered feasible subsequent to professional assessment. Patients categorized as possible and probable to benefit from PFO closure surgery by PASCAL system experience a reduced risk of postoperative late-onset atrial fibrillation (>45 days) and stroke recurrence (2).

After 2017, four randomized controlled; CLOSE (4), RESPECT (74), REDUCE (5), DEFENSE-PFO (75) uncovered that transcatheter closure of PFO was more effective than medical therapy alone. Another meta-analysis found that the annualized risk of recurrent stroke was approximately 1% in patients treated with medical therapy alone, whereas that of recurrent stroke was decreased by approximately 60% in patients undergoing transcatheter PFO closure (32). Considering the available evidence regarding the effectiveness of transcatheter PFO closure, the 2022 SCAI Guidelines for Management of Patent Foramen Ovale strongly recommend the use of transcatheter PFO closure in patients aged between 18 and 60 years with previous PFO-AS. PFO closure can effectively reduce the risk of stroke recurrence, regardless of the degree of PFO anatomical complexity. This suggests that the benefits of PFO closure extend to both high-risk and low-risk PFO patients. It’s important to note that most patients included in current studies are under the age of 60, limiting the generalizability of the findings to all age groups. It has been shown (76) that transcatheter closure of PFO may be as safe and effective in patients >60 years of age as in patients <60 years of age. A recent study in 2024 found (77) 143 patients with PFO closure and 199 patients with drug-only stroke aged >60 years, with a mean follow-up of 5.6 ± 1.5 years. All patients who did not undergo PFO closure exhibited persistent shunts. In contrast, only seven of 134 patients who received PFO closure had residual shunts. Additionally, the rates of new-onset atrial fibrillation, recurrent stroke, unexplained death, and neurological death were 5, 6%, 1, and 2, respectively, in the group without closure, compared to 3, 3%, 0, and 0 in the closure group. This study suggests that closure therapy should be adopted in patients older than 60 years as in younger patients. However, elderly patients have a higher incidence of postoperative complications such as stroke, TIA, peripheral embolism, and postoperative atrial fibrillation (78). Additional prospective studies are necessary to evaluate the safety and efficacy of transcatheter PFO closure in patients younger than 18 and older than 60 years of age.

However, Park J et al. (79) performed a retrospective study in JASE comprising 4,804 patients with a history of HF, of whom 981 patients with PFO, 161 underwent PFO occlusion. During the 3.5-year follow-up, the incidence of HF in patients with PFO was lower than that in patients without PFO, and the rate of HF in patients who received PFO occlusion was higher. Moreover, recent research also suggests that PFO closure may increase the risk of Heart Failure (HF) with preserved Ejection Fraction (HFpEF) (80). On the one hand, before performing occlusion surgery, PFO may act as a “pressure reducing valve” for high-risk populations (patients with concomitant structural heart disease, left atrial enlargement, left ventricular hypertrophy, etc.), allowing left to right shunting during exercise or elevated left atrial pressure, reducing the risk of pulmonary congestion; On the other hand, it may be due to the reduced left atrial compliance after occlusion surgery, which weakens the protective effect of natural shunting. Although this study does not alter the indications for PFO occlusion, clinicians must consider the hemodynamic implications of the procedure. Ideal candidates for PFO occlusion are likely true PFO-Associated Stroke patients without underlying structural or functional heart disease. Preclinical studies suggest that these patients have a lower risk of developing heart failure following PFO closure. In patients with structural or functional heart diseases, PFO closure can reduce the incidence of stroke, but it will also increase the risk of HF. Therefore, a large-scale prospective study is needed to explain the relationship between PFO and HF, so that for patients with PFO-AS, we can more fully confirm the indications of PFO occlusion and increase long-term prognosis benefits.

In summary, the effectiveness of PFO occlusion in reducing the risk of recurrent stroke has been confirmed in randomized controlled trials, approved by guidelines and recommended at this level (3, 73, 81) (Table 3). The subset of patients show may optimally benefit from transcatheter PFO closure is currently not fully understood, and the 2022 SCAI still recommend a RoPE score ≥ 7 (3). Future research should focus on a multi-dimensional approach, considering PFO anatomical and morphological characteristics, patient history (particularly the presence of structural and functional heart diseases), various diagnostic techniques, and scoring systems. This comprehensive approach will enable the identification of specific patient subgroups who may benefit most from PFO closure and facilitate personalized diagnosis and treatment.

**Table 3:** Guidelines for transcatheter PFO closure therapy.

	2020AAN guidelines	2021AHA/ASA guidelines	2022SCAI guidelines
Suggestions	For patients under 60 years of age who have no other mechanism of stroke and have no embolic embolism and PFO, the clinician can weigh the potential benefits and risks before performing PFO closure surgery	In patients aged 18 to 60 years with non-lacunar ischemic stroke, where the cause is unknown despite thorough evaluation and PFO has high-risk anatomical features, transcatheter device closure of PFO+ long-term antiplatelet therapy is more reasonable than antiplatelet therapy alone to prevent stroke recurrence	For patients aged 18 to 60 years with prior PFO-related stroke, transcatheter PFO closure is recommended compared with antiplatelet therapy, which is superior to antiplatelet therapy alone, regardless of the patient’s anatomy; In patients with AF with a history of ischemic stroke, PFO is closed by a compensation cabinet
Recommendation, level of evidence	C	2a	Strong recommendation

There are two methods of transcatheter PFO occlusion and transcatheter PFO suture before occlusion.

##### 4.2.2.2 Transcatheter closure of PFO

Currently, two types of occluders are available, one is metal occluders related to nitinol, and the other is biodegradable occluders.

The ever-increasing clinical demand and technological progress have resulted in the widespread use of Amplatzer and IrisFIT occluders in clinical practice as polyester fibers to facilitate the positioning of the occluders toward the interatrial septum so that it is unaffected by the spatial orientation of the interatrial septum itself. However, nickel-titanium occluders are associated with long-term heart problems: such as nickel ion precipitation and nickel allergy, mechanical complications of long-term wear (such as erosion, perforation, cardiac tamponade), displacement, embolism and serious postoperative residual shunt problems (82). To determine the most suitable occluders for patients, the commonly used reference indicators include: (1) Age: in principle, 18 mm occluders should be selected for ages below 18 years; (2) Combined ASA: the occluders should be covered (pay attention to the activity of atrial septum); (3) Select the appropriate size occluders with reference to the PFO size and the distance between the upper and lower cavities in combination with TEE results (Table 4).

**Table 4:** The results of TEE for PFO occluders size selection’s reference.

The shortest distance from the defect to the root of the aorta or from the defect to the superior vena cava orifice (mm)	Dimensions (mm)
<9 mm	Implant
9–12.4 mm	18
12.5–14.9	25
15–17.4	30
≥17.5 mm	35

Biodegradable occluders include partially biodegradable occluders and fully biodegradable occluders. Biodegradable occluders are more technically demanding to fabricate, require many material and specific PFO sizes and location. Therefore, when performing PFO closure, clinicians should accurately measure and display the location, size, tunnel length and distance from the surrounding tissue of the PFO, and then select the appropriate size of the occluders. Currently, few degradable occluders products are available in the market, and their clinical effects need to further investigated.

##### 4.2.2.3 Transcatheter PFO suture

The PFO stapler is currently under development, and the Noble Stitch stapler consists of three dedicated catheters, two of which suture delivery catheters capture and suture the secondary septum and the primary septum, respectively, and then secure and trim the excess suture using another Kwik not catheter. Compared to the PFO occluders, the PFO stapler lacks a permanent prosthesis, minimizes the incidence of atrial fibrillation, and since it is metal-free, elicits no metal allergies, and does not require long-term antiplatelet therapy. Studies have shown that RLS ≥ Grade II, width > 5 mm, and length < 10 mm before PFO surgery are independent predictors of residual shunt ≥ Grade II after transcatheter closure of PFO (83). A multicenter, prospective Noble Stitch-based study of 186 PFO patients who had preoperative RLS ≥ grade II and were followed up for an average of (206 ± 130) days after surgery found that 75% of patients achieved complete closure (RLS grade 0) and 89% had RLS ≤ grade I without suture-related complications. A non-randomized, open-label NobleStitch EL STITCH trial with a larger sample size and longer follow-up is currently underway. The objective of the trial is to compare efficacy of the PFO closure and reduce the incidence of ischemic stroke events between NobleStitch and Amplatzer PFO. In 2025, The Shenzhen Hospital of Fuwai Hospital Chinese Academy of Medical Sciences will initiate a clinical trial on “A multicenter, randomized controlled, non-inferiority study to evaluate the safety and effectiveness of transcatheter PFO stapler system.” The study will use the HaloStitch transcatheter PFO stapler system. Compared with metal occluders, HaloStitch also has no nickel ion precipitation, cardiac abrasion, postoperative atrial fibrillation and other complications, and does not lead to long-term anticoagulation.

Both traditional transcatheter PFO closure and transcatheter PFO suture require a personalized approach tailored to the patient’s individual circumstances, including their medical condition, economic factors, potential postoperative complications, residual shunts, and other relevant factors.

##### 4.2.2.4 Post closure drug-treatment

In consonance with pertinent guidelines (3), for individuals categorized as low-risk, the administration of DAPT, consisting of aspirin (75–100 mg/day) in conjunction with clopidogrel (75 mg/day), is advocated for a duration of 3–6 months. Subsequently, a transition to monotherapy, typically with aspirin, is recommended for long-term maintenance, with a minimum duration of 12–24 months subsequent to the surgical procedure. For patients who manifest concurrent atrial fibrillation or other indications for anticoagulation, or who exhibit anticoagulation status or a history of recurrent thrombotic events, anticoagulation therapy should be considered in light of individual circumstances and assessed bleeding risks, with the treatment course abbreviated to the greatest extent feasible. Moreover, in cases of significant postoperative occluders thrombosis or residual shunt, the duration of dual antiplatelet therapy may be prolonged or a switch to anticoagulant therapy may be warranted. For patients at high risk of bleeding, the duration of DAPT may be reduced to 1–3 months, or aspirin monotherapy may be employed.

##### 4.2.2.5 Post closure assessment

According to pertinent guidelines (3), echocardiography and ECG should be performed at 24 h, 1, 3, 6, 1 2 months and yearly after PFO closure, and TTE right heart contrast echocardiography or c-TCD should be performed when necessary. Imaging assessments including occluders position, presence or absence of occluders thrombus, and changes in cardiac structure. TTE right heart contrast echocardiography or c-TCD should be performed 6 months after operation to determine whether there is still existing RLS. For patients with a significant number of RLS, regular follow-up was continued, with repeat TTE right heart contrast echocardiography or c-TCD performed after one year. If RLS persisted, a TEE was recommended. If any clinical symptoms are detected during the entire process, timely ECG and echocardiography are recommended to identify new complications, such as arrhythmia and new thrombus. Moreover, the prognoses of the patients need to be comprehensively assessed in combination with the relevant scoring scales such as clinical symptoms, postoperative NIHSS and mRS.

## 5 Conclusion and outlook

Current research has focused on investigating the pathogenesis, clinical characteristics, acute treatment and secondary prevention of PFO-AS.

Patients with PFO-AS require a treatment approach similar to that of CS during the acute phase. However, their younger age and milder symptoms necessitate a more targeted treatment strategy. PFO-AS patients often have fewer underlying comorbidities, allowing for greater flexibility in thrombolytic timing, drug selection, adjuvant therapies, mechanical thrombectomy, and bridging therapy prior to thrombectomy. Similarly, in terms of secondary prevention, the advantages of occlusion therapy have been documented. However, clinicians should consider the patient’s history of heart disease and assess whether the patient presents a clear structural and functional heart disease as well as perform a comprehensive evaluation of the PFO structure (channel length and opening diameter), shunt volume, blood flow velocity and thrombus size, thrombus site, vascular occlusion and other clinical data in combination with the commonly used PFO screening methods and imaging related data, and select the appropriate treatment plan to prevent the occurrence and recurrence of various vascular events.

Unlike for stroke patients with known etiology, circulating biomarkers and genetic factors should be used to determine the etiology of PFO-AS patients. In future, researchers should explore PFO-associated circulating metabolites and causative genes, to identify new targets for the diagnosis and treatment of PFO-associated diseases.
